# *Coptis chinensis* Franch. Suppresses Invasive Pulmonary Aspergillosis by Augmenting NADPH-Dependent Neutrophil Extracellular Traps via Dual Modulation of Complement Activation and Gut Microbiota

**DOI:** 10.3390/cimb48040424

**Published:** 2026-04-20

**Authors:** Zhuqiao Jiang, Lingmei Zhou, Jinping Wang, Hao Sun, Liwen Cai, Hanqi Yin, Hui Zhu, Ming Li, Zhuoya Wang

**Affiliations:** 1 Guangdong Provincial Key Laboratory of Pharmaceutical Bioactive Substances, School of Basic Medical Sciences, Guangdong Pharmaceutical University, Guangzhou 510006, China; 2 South China Institute of Biomedicine, Guangzhou 510535, China; 3 The First Affiliated Hospital, Guangdong Pharmaceutical University, Guangzhou 510080, China

**Keywords:** *Coptis chinensis* Franch., invasive pulmonary aspergillosis, neutrophil extracellular traps, NADPH oxidase, complement activation

## Abstract

Invasive pulmonary aspergillosis (IPA) poses a serious threat to immunocompromised hosts, with limited therapeutic options highlighting the need for novel strategies. *Coptis chinensis* Franch. (CCF), a traditional Chinese herb containing antimicrobial alkaloids like berberine, was investigated for its therapeutic efficacy and immunological effects in a murine IPA model. Immunosuppressed female KM mice infected with *Aspergillus fumigatus* AF293 were treated with CCF or amphotericin B (AmB). CCF significantly improved survival, reduced fungal burden, and alleviated lung pathology, without inducing hepatotoxicity or nephrotoxicity. Transcriptomic profiling revealed a time-dependent immune response. Complement-related pathways were enriched at 2 days post-infection, whereas neutrophil recruitment and NET-related pathways became more prominent by day 4. Hub gene analysis identified Syk, Rac2, Ncf1, and Cybb as key nodes associated with the NADPH oxidase complex. Western blot and inhibitor experiments further supported the involvement of this pathway in CCF-mediated protection. Additionally, 16S rDNA sequencing indicated enrichment of *Clostridium* species in the gut microbiota of CCF-treated mice, which was positively correlated with the expression of NADPH oxidase-related genes, suggesting a potential gut–lung association. In conclusion, these findings support the antifungal efficacy of CCF in IPA and suggest that its protective effects may involve coordinated changes in complement-related responses, NADPH oxidase-associated neutrophil activity, and gut microbiota composition.

## 1. Introduction

Invasive pulmonary aspergillosis (IPA), predominantly caused by *Aspergillus fumigatus* (*A. fumigatus*), is a life-threatening fungal infection that commonly affects immunocompromised patients undergoing chemotherapy, organ transplantation, or immunosuppressive therapy [1,2]. Despite the availability of antifungal agents such as amphotericin B (AmB) and voriconazole, the clinical management of IPA remains suboptimal due to dose-limiting toxicities, increasing antifungal resistance, and unsatisfactory long-term outcomes [1,3,4]. Thus, there is a critical need to explore novel antifungal therapies with improved safety profiles and multifaceted mechanisms of action.

Traditional Chinese medicine, with its long-standing history in treating infectious and inflammatory diseases, has attracted growing interest as an alternative therapeutic strategy [5]. Among these, *Coptis chinensis* Franch. (CCF), a well-characterized herbal remedy, exerts broad pharmacological activities including antimicrobial, anti-inflammatory, and immunomodulatory effects, mainly attributed to its major alkaloid, berberine [5,6,7,8]. While in vitro studies have suggested antifungal potential [9], the in vivo efficacy of CCF against IPA and its associated immunological mechanisms remain largely unexplored.

Recent advances in fungal immunology have highlighted the importance of coordinated immune activation, where early innate sensing pathways such as the complement system facilitate downstream effector responses including neutrophil recruitment, NADPH oxidase activation, and neutrophil extracellular trap (NET) formation [10,11,12]. However, whether such temporally ordered immune cascades can be modulated pharmacologically, particularly by traditional herbal agents, remains unclear.

Additionally, previous studies have reported that CCF can modulate gut microbiota composition in various disease models, including metabolic and inflammatory disorders [13,14,15]. However, whether CCF exerts similar microbiota-modulating effects in the context of IPA remains unknown. Furthermore, it is unclear whether such microbiota alterations, if present, contribute to host antifungal immunity via redox-sensitive immune pathways.

To address these gaps, this study aimed to evaluate the therapeutic efficacy and safety of CCF in a murine model of invasive pulmonary aspergillosis and to investigate the immune changes related to its protective effects. We focused on whether CCF treatment was linked to an early complement-related response, later neutrophil- and redox-related changes, and altered gut microbiota composition. This study combines in vivo efficacy and safety evaluation with time-resolved transcriptomic analysis, pharmacological validation, and microbiota profiling in a single model. This design provides a broader view of how CCF may influence host immune responses and gut microbiota in IPA.

## 2. Materials and Methods

### 2.1. Materials

Crude slices of CCF were purchased from Guangdong Provincial Hospital of Chinese Medicine. Cyclophosphamide, cortisone acetate, AmB, palmatine, coptisine, berberine, epiberberine and jatrorrhizine were purchased from Shanghai Macklin Biochemical Co., Ltd. (Shanghai, China).

### 2.2. Fungal Strains and Collection of Resting Conidia

The *A. fumigatus* strain AF293 used in this study was provided by the Department of Medical Laboratory, Zhujiang Hospital of Southern Medical University. The strain was preserved as glycerol stocks at −80 °C for long-term storage. Prior to use, the strain was revived in Sabouraud Dextrose Broth (SDB) at 37 °C for 48 h with shaking, followed by inoculation onto Sabouraud Dextrose Agar (SDA) and incubation at 37 °C for 72 h for conidial development. Conidia were harvested by washing the culture with 10 mL of PBS containing 0.1% Tween 80, filtering through 8 layers of sterile gauze, and repeating the washing to ensure purity. The filtrate was examined under a microscope to confirm the presence of predominantly conidia. This standardized protocol of fresh harvest, filtration, and microscopic examination is designed to yield a conidial suspension of high purity and viability for murine infection. The conidial suspension was centrifuged at 800× *g* for 10 min, and the pellet was resuspended in PBS. The concentration of conidia was determined using a hemocytometer and adjusted to the desired level with PBS containing 0.1% Tween 80. The freshly prepared conidial suspension was used for infection immediately.

### 2.3. Preparation of CCF Extract

After soaking in cold distilled water for 2 h (1:10, *w*/*w*), the CCF slices were boiled for 30 min in a decoction pot. The liquid was collected, and the dregs were added to cold water and boiled again for 30 min. These two decoctions were combined and concentrated to a concentration of 1 g/mL using a rotary evaporator (BK-RE-1A, Jinan Olabo Technology Co., Ltd., Jinan, China). The concentrated CCF decoction was sterilized by filtration through a 0.22 μm filter (MilliporeSigma, Burlington, MA, USA) and stored at −20 °C until use. To minimize batch-related variability, all key experiments in this study were performed using a single, well-characterized batch of CCF extract, and its chemical profile was verified by HPLC before use. High-performance liquid chromatography (HPLC) revealed the presence of palmatine, coptisine, berberine, epiberberine, and jatrorrhizine in the CCF decoction (Figure S1, Table S1). These alkaloids are recognized bioactive constituents known for their antimicrobial and immunomodulatory properties, as documented in previous studies focusing on related botanical sources or similar compounds [16]. This compositional profile indicates the suitability of the extract for evaluating its antifungal activity against *Aspergillus* and investigating its immunomodulatory effects.

### 2.4. Mice

Female KM mice (7–8 weeks old, 26 ± 2 g) were purchased from Guangdong Medical Laboratory Animal Center (Foshan, China, License No. SYXK (Yue) 2022-0002). Mice were housed under specific pathogen-free (SPF) conditions in the Animal Facility of Guangdong Pharmaceutical University (Guangzhou, China, License No. SYXK (Yue) 2022-0125), with a controlled environment (22 ± 2 °C, 50–60% humidity, 12-h light/dark cycle). All experiments involving animals were approved by the Guangdong Pharmaceutical University Laboratory Animal Ethics Committee (Approval No. gdpulac2020086). At the end of experiments, all mice were euthanized by CO_2_ inhalation anesthesia followed by cervical dislocation, ensuring minimal suffering.

### 2.5. Immunosuppression and Infection

To induce immunosuppression, all mice received cyclophosphamide (200 mg/kg, intraperitoneally) and hydrocortisone acetate (250 mg/kg, subcutaneously) at −2 dpi and +3 dpi. On the day of infection (0 dpi), mice were intranasally inoculated with *A. fumigatus* conidia to establish the IPA model. Infection cages were prepared following the method of Donald C. Sheppard [17]. Mice were placed in the infection cage inside a biological safety cabinet, and 12 mL of a 1 × 10^9^ conidia/mL suspension was nebulized using an ultrasonic nebulizer (403G2, aiHUjia, Yancheng, China) for 1 h. Starting at +1 dpi, drug treatments were administered based on group assignments. Each group consisted of 10 KM mice, with survival monitored from 0 to +7 dpi and drug treatments initiated at +1 dpi. Right and left lung tissues were collected at +2, +4, and +6 dpi for CFU and 18S rDNA analysis, respectively. Tissues were homogenized, diluted, and processed, with CFU plated on SDA and 18S rDNA analyzed using qPCR.

### 2.6. Sample Collection and Tissue Allocation

At +2, +4, and +6 days post-infection, surviving mice were anesthetized and euthanized according to the approved animal protocol before sample collection under aseptic conditions. Lung tissues, fecal samples, and plasma were harvested. For standardized tissue allocation, the left lung was mainly used for 18S rDNA quantification and RNA-seq, while a designated portion was fixed for histopathological analysis. Two anatomically fixed lobes of the right lung were subjected to CFU analysis, and the remaining right lung tissue was stored for Western blot and qPCR.

### 2.7. Effect of CCF on IPA Survival

To assess the therapeutic efficacy and systemic effects of CCF in IPA, survival and body weight were monitored following infection. Forty KM mice were randomly assigned to four groups (*n* = 10 per group) using a computer-generated randomization sequence: an immunocompetent infected control group (CN), an IPA group (immunosuppressed and untreated), an AmB treatment group (10 mg/kg, intraperitoneally), and a CCF treatment group (455 mg/kg, oral gavage). No formal a priori power calculation was performed. Sample sizes were selected with reference to prior murine invasive pulmonary aspergillosis studies with comparable survival and fungal-burden endpoints, many of which used groups of approximately 10 mice, together with our pilot observations, while aiming to minimize animal use [18]. The doses of CCF decoction and AmB in this study adhered to the clinical recommendations of the *Pharmacopoeia of the People’s Republic of China* (ChP, 2025 edition) [19]. Using body surface area normalization with a human-to-mouse conversion factor of 12.3, the mouse doses of 455 mg/kg (CCF) and 10 mg/kg (AmB) correspond to a human equivalent dose (HED) of approximately 2.2 g and 0.05 g, respectively, for a 60 kg adult. Mice in the IPA, AmB, and CCF groups received immunosuppressive treatment prior to infection, while mice in the CN group remained immunocompetent and were infected with *A. fumigatus* on the same day (+0 dpi) to serve as a non-immunosuppressed infection control. Survival was monitored daily from day 0 to day 7 post-infection to evaluate treatment-related mortality. In parallel, body weight was recorded daily from day −2 to day +6 to assess general health status. At +2, +4, and +6 dpi, surviving mice were sampled for downstream analyses: lung tissues, fecal samples, and plasma were collected under aseptic conditions. To standardize tissue allocation and account for attrition due to mortality, the entire left lung was dedicated to 18S rDNA quantification and RNA-seq; prior to nucleic acid extraction, a defined portion of the left lung was fixed, paraffin-embedded, and sectioned for histopathology. Two fixed-position lobes of the right lung were used for colony-forming unit (CFU) analysis, and the remaining right-lung tissue (and other organs as needed) was reserved for Western blot and qPCR.

### 2.8. Colony Forming Unit (CFU) Analysis

Right lung tissues were collected at +2, +4, and +6 dpi under sterile conditions. Samples from the upper, middle, and lower lobes of the right lung were weighed and placed in 200 µL of sterile saline. The tissues were homogenized using a cryogenic tissue grinder with grinding beads, and then 800 µL of sterile saline was added for a 5-fold dilution. Next, 100 µL of the homogenate was plated on SDA in triplicate. Plates were incubated at 37 °C for 24 h, and colony-forming units (CFU) were counted to determine fungal burden. CFU was calculated using the following formula: CFU/g = (average number of colonies from three plates × 10)/tissue weight. CFU counting was performed by two independent investigators blinded to group allocation.

### 2.9. 18S rDNA Quantification

Left lung tissues were collected at +2, +4, and +6 dpi under sterile conditions. DNA was extracted using the TIANamp Genomic DNA Kit (DP304, TIANGEN Biotech, Beijing, China) with modifications: 200 µL GA Buffer and 0.5 mm glass beads were added, followed by homogenization at 4 °C at 21 m/s for 5 min using a cryogenic tissue grinder. Subsequent steps followed the manufacturer’s instructions. Fungal burden was quantified using real-time qPCR targeting the region of the ribosomal subunit gene of each Aspergillus species, as described previously, with the TB Green^®^ Premix Ex Taq™ II (Tli RNaseH Plus) qPCR Kit (Takara, Kusatsu, Japan). The primer and probe sequences used in this study are listed in Table S2. qPCR was performed using 100 ng of total lung DNA from each homogenized sample as the template. Amplification and melt curves were verified, and mouse β-actin was used as the internal reference gene. Relative quantification was calculated using the 2^−ΔCt^× 1000 method for intergroup comparisons.

### 2.10. Histopathological Analysis

At +6 dpi, left lungs were collected from each group for analysis. The tissues were fixed in 4% paraformaldehyde, embedded in paraffin, and sectioned. Sections were stained with hematoxylin–eosin (HE) and Gomori methenamine silver (GMS) by Hubei BIOSSCI Biotechnology Co., LTD. (RR820A, Kusatsu, Japan). Stained sections were scanned using the NanoZoomer^®^ S360 (Hamamatsu Photonics K.K., Hamamatsu, Japan) for brightfield imaging. Images were captured under consistent illumination and camera settings to evaluate pathological differences among the groups. Histopathological evaluation was performed with blinded evaluation by two independent investigators to ensure analytical consistency.

### 2.11. Biochemical Assessment

At +2, +4, and +6 dpi, five mice per group were randomly selected and anesthetized with isoflurane. Blood was collected via retro-orbital bleeding, clotted at room temperature for 30 min, and centrifuged at 3000 rpm for 10 min at 4 °C. Serum was separated, flash-frozen in liquid nitrogen, and stored at −80 °C. ALT and AST levels were measured using alanine aminotransferase (C009-2-1) and aspartate aminotransferase (C010-2-1) assay kits, respectively, while BUN and CRE levels were determined using urea nitrogen (C013-2-1) and creatinine (C011-2-1) assay kits (Nanjing Jiancheng Bioengineering Institute, Nanjing, China). All assays followed the manufacturer’s instructions, and absorbance was measured at the specified wavelength using a Multiskan SkyHigh Microplate Spectrophotometer (Thermo Fisher Scientific, Waltham, MA, USA). Results were compared with the naïve (uninfected, untreated) control group to evaluate potential hepatotoxic or nephrotoxic effects of the treatments.

### 2.12. RNA-Seq Analysis and CCF-Specific DEG Identification

For RNA-Seq, three biological replicates per group per time point (+2, +4, and +6 dpi) were used for library construction and sequencing. Total RNA was extracted from lung tissue samples using TRIzol reagent (Invitrogen, Waltham, MA, USA) following the manufacturer’s instructions. RNA concentration and purity were assessed using a NanoPhotometer spectrophotometer (Implen, Munich, Germany), and integrity was evaluated with an Agilent 2100 Bioanalyzer (Agilent Technologies, Santa Clara, CA, USA). Only RNA samples with an RNA integrity number (RIN) ≥ 7.0 were selected for library construction. mRNA was enriched from total RNA using Oligo(dT) magnetic beads, fragmented, and reverse-transcribed into cDNA. First-strand synthesis was performed using random hexamer primers, followed by second-strand synthesis using DNA polymerase I and RNase H. The resulting double-stranded cDNA underwent end repair, A-tailing, adaptor ligation, and PCR amplification. Purified libraries were quantified using a Qubit 2.0 Fluorometer (Thermo Fisher Scientific, Waltham, MA, USA) and quality-checked with an Agilent 2100 Bioanalyzer. Sequencing was conducted on an Illumina NovaSeq 6000 platform (paired-end, 150 bp) by Guangdong Pangu Medical Technology Co., Ltd. (Guangzhou, China). Raw sequencing reads were quality-trimmed using Trimmomatic to remove adapters and low-quality bases. Clean reads were aligned to the mouse reference genome (GRCm38) using HISAT2 (version 2.2.1), and gene-level quantification was performed with featureCounts. Differentially expressed genes (DEGs) were identified using DESeq2. Given the time-resolved and comparative design of the RNA-seq analysis, genes with a nominal (uncorrected) *p* value < 0.05 were retained for exploratory screening of candidate pathways and treatment-related transcriptional changes. To define CCF-specific DEGs, pairwise comparisons were conducted between the CCF vs. IPA and CCF vs. AmB groups at each time point (+2, +4, and +6 dpi). Genes that were significantly and consistently differentially expressed in both comparisons (same direction of regulation) were retained as CCF-specific DEGs. Venn diagrams were used to visualize overlapping gene sets. These CCF-specific DEGs were subsequently subjected to Kyoto Encyclopedia of Genes and Genomes (KEGG) pathway enrichment analysis to elucidate their potential biological functions.

### 2.13. Weighted Gene Co-Expression Network Analysis

Weighted gene co-expression network analysis (WGCNA) was conducted using the WGCNA R package (v1.47) to identify gene modules associated with CCF treatment in IPA. After filtering genes with low expression or variance, expression data were normalized by variance-stabilizing transformation. A soft-thresholding power of 9 was selected based on the scale-free topology criterion, as it was the lowest power at which the scale-free topology fit index reached a plateau above 0.9. An unsigned adjacency matrix was computed from pairwise Pearson correlations and transformed into a topological overlap matrix (TOM) to measure gene interconnectedness. Gene clustering was performed based on TOM dissimilarity, and modules were defined using the dynamic tree cut algorithm with the parameter minModuleSize = 50. Closely related modules were merged using a correlation threshold of 0.85 (mergeCutHeight = 0.15). A total of 13,539 genes were clustered into 20 co-expression modules. Module eigengenes (MEs) were calculated and correlated with clinical traits, including treatment group, infection time point, CFU counts, and *A. fumigatus* 18S rDNA expression. Genes with high module membership (MM), gene significance (GS), and intramodular connectivity (K.in) were considered candidate hub genes. Network visualization was performed using Cytoscape (v3.3.0). GO and KEGG enrichment analyses were performed for each module using hypergeometric testing. Multiple testing correction was applied using the Benjamini–Hochberg method, and terms or pathways with FDR < 0.05 were considered significantly enriched.

### 2.14. Bayesian Network Analysis

To investigate potential regulatory hierarchies underlying CCF-mediated immune responses, we conducted Bayesian network analysis based on time-resolved RNA-Seq data. DEGs were first identified within each treatment group by pairwise comparisons between +2 dpi vs. +4 dpi and +4 dpi vs. +6 dpi. The intersecting DEGs across these two time points were selected to ensure temporal continuity of expression changes and used as the input gene set for Bayesian network construction. Bayesian inference was performed to evaluate conditional dependencies among gene pairs, allowing the probabilistic reconstruction of gene regulatory interactions. Only edges (gene–gene connections) with a conditional probability > 0.6 were retained to ensure high-confidence regulatory relationships. For each gene, the degree of connectivity (DEGREE) was calculated as the number of direct regulatory interactions. Genes in the top 10% by DEGREE score were defined as hub genes and considered key regulatory nodes. Genes with DEGREE > 6 were visualized at the core of the network.

### 2.15. qPCR

Total RNA was extracted from lung tissues using the FastPure Cell/Tissue Total RNA Isolation Kit V2 (Vazyme, Nanjing, China) in an RNase-free environment to prevent RNA degradation. Reverse transcription was performed to synthesize cDNA from 1 µg of total RNA using the PrimeScript™ RT Reagent Kit with gDNA Eraser (Takara, Kusatsu, Japan). Quantitative real-time PCR (qPCR) was conducted on the CFX96 Real-Time PCR System (Bio-Rad Laboratories, Hercules, CA, USA) using TB Green^®^ Premix Ex Taq™ II (Tli RNaseH Plus) (Takara, Kusatsu, Japan). Each reaction contained 100 ng of cDNA and gene-specific primers, with primer and probe sequences detailed in Table S2. All reactions were carried out in triplicate, using β-actin as the reference gene for normalization. Relative gene expression was determined using the 2^−ΔΔCt^ method. The sequences of all primers used for qPCR are listed in Supplementary Table S2.

### 2.16. Western Blot

Total protein was extracted from 10–20 mg of mouse lung tissue using 200 µL of RIPA lysis buffer (P0013B, Shanghai Beyotime Biotechnology Co., Ltd., Shanghai, China) with EDTA-free protease inhibitor (05892791001, Roche, Basel, Switzerland) in an RNase-free environment. The tissue was homogenized, centrifuged at 12,000× *g* for 10 min at 4 °C, and the supernatant collected. Protein extracts were initially assessed by microvolume A280 measurement on a NanoDrop 2000 (Yomim, Shanghai Yuyan Instruments Co., Ltd., Shanghai, China) as a rapid QC check, with the instrument blanked using the same RIPA working buffer. Because RIPA can interfere with A280-based quantification, final protein concentrations used for all downstream analyses were determined using a bicinchoninic acid (BCA) assay (P0009, Shanghai Beyotime Biotechnology Co., Ltd., Shanghai, China) with BSA standards (standard curve R^2^ = 0.9988; read at 562 nm). Based on the BCA-derived concentration, a 30 µg protein sample was mixed with 5× Loading Buffer, denatured by heating, and stored at −80 °C. Proteins were separated on 10% SDS-PAGE gels using an electrophoresis system (Bio-Rad, Hercules, CA, USA) at 120 V, then transferred to PVDF membranes using a transfer system (Bio-Rad, USA) at 200 mA, and blocked with 5% non-fat dry milk in PBST for 1 h at room temperature. Membranes were incubated with primary antibodies overnight at 4 °C, washed with PBST (5 times, 5 min each), then incubated with secondary antibodies for 1 h at room temperature and washed again. Protein bands were visualized using an automated chemiluminescence imaging system (Shanghai Tanon Science & Technology Co., Ltd., Shanghai, China) and quantified with ImageJ software (version 1.53t, National Institutes of Health, Bethesda, MD, USA).

### 2.17. Antibodies

Primary antibodies used in Western Blot included Beta Actin Monoclonal antibody (66009-1-Ig, Proteintech, Wuhan, China), NCF4/p40phox Rabbit pAb (A2096, Abclonal, Wuhan, China), Anti-NOX2/gp91phox Recombinant Rabbit Monoclonal Antibody (ET1611-44, Huabio, Hangzhou, China), RAC2 Rabbit pAb (A1139, Abclonal, China) and Anti-NCF1/p47phox Rabbit pAb (GB11724, Servicebio, Wuhan, China). Detection was performed using HRP-conjugated Goat anti-Rabbit IgG (H+L) (SA00001-2, Proteintech, China) and HRP-conjugated Goat anti-Mouse IgG (H+L) (SA00001-1, Proteintech, China), both diluted in PBST as per the manufacturer’s recommendations.

### 2.18. Effect of Rhizoma Coptidis on NADPH Oxidase, Survival, and Fungal Burden

To assess whether CCF modulates NADPH oxidase activity and impacts host survival, we conducted an experiment with four groups: control, CCF, CCF + Apocynin, and Apocynin alone. Mice received daily treatments of CCF (455 mg/kg by gavage), Apocynin (50 mg/kg intraperitoneally), CCF + Apocynin (combined treatment), or vehicle (control). Apocynin (50 mg/kg, i.p., once daily) was used as a pharmacological probe of oxidative pathways. The dose and route were selected based on prior murine studies using apocynin to modulate NADPH oxidase/ROS-related responses in vivo [20]. To ensure systemic exposure during the relevant oxidative-response window, apocynin was administered 30 min before CCF gavage from +1 dpi to +8 dpi. Survival was monitored from 0 to +8 dpi, and lung fungal burden was assessed at +4 dpi by counting CFUs from lung homogenates plated on SDA and incubated at 37 °C for 48 h. Statistical analyses were performed using the Log-rank test for survival and one-way ANOVA for CFU count comparisons.

### 2.19. 16S rDNA Sequencing and Microbiota Analysis

Fecal samples were collected from mice at 7 days post-infection using stress-induced defecation, immediately snap-frozen in liquid nitrogen, and stored at −80 °C until further analysis. Total DNA was extracted using the TIANamp Stool DNA Kit (TIANGEN, Beijing, China; Cat. No. DP328) according to the manufacturer’s instructions. To monitor contamination, extraction blank controls (no template) were processed alongside each batch of samples. Full-length 16S rRNA genes were amplified using universal primers 27F and 1492R with PacBio-specific barcodes. PCR amplification was performed with KAPA HiFi Hot Start DNA Polymerase (KAPA Biosystems, Wilmington, DE, USA), including PCR blank controls. This was followed by quality control using the Fragment Analyzer (Agilent Technologies, Santa Clara, CA, USA). Amplicons were pooled at equimolar concentrations and processed using the SMRTbell Express Template Prep Kit 2.0 (PacBio, Menlo Park, CA, USA). Sequencing was performed on the PacBio Sequel II platform by Berry Genomics (Beijing, China). Raw sequencing data were processed using SMRTlink and QIIME2 pipelines. Reads were demultiplexed and filtered based on length and primer content. Amplicon sequence variants (ASVs) were generated using DADA2 for denoising. Taxonomic assignment of representative ASVs was performed using a naïve Bayesian classifier against the SILVA reference database, with removal of mitochondrial and low-confidence (<10 reads) ASVs. Species abundance tables were constructed across taxonomic levels (phylum to species) and used for downstream diversity and compositional analyses. Alpha diversity indices (Chao1, Shannon, Simpson, and Goods_coverage) were calculated using QIIME2 to evaluate species richness and diversity. Beta diversity was assessed based on Bray–Curtis distances and visualized through principal coordinate analysis (PCoA). Differential abundance analysis between groups was conducted using LEfSe (LDA score > 2.0) and metagenomeSeq (*p* < 0.05), incorporating Kruskal–Wallis and Wilcoxon tests, as well as zero-inflated Gaussian modeling.

### 2.20. Statistical Analysis

Statistical analyses were performed using GraphPad Prism (version 9.3.1) and R (version 4.5.0). Data are presented as mean ± standard deviation (SD). One-way ANOVA followed by Tukey’s post hoc test was used for multiple-group comparisons. Survival data were analyzed using the Kaplan–Meier method and compared using the log-rank (Mantel–Cox) test. Correlation analyses were performed using Spearman’s rank correlation. For RNA-seq analysis, differential expression was performed using DESeq2 as described above, and the resulting DEG set was used for exploratory pathway and network analyses. For other statistical analyses, a *p* value < 0.05 was considered statistically significant.

## 3. Results

### 3.1. CCF Improves Survival and Reduces Fungal Burden in IPA Mice

**Figure 1:**
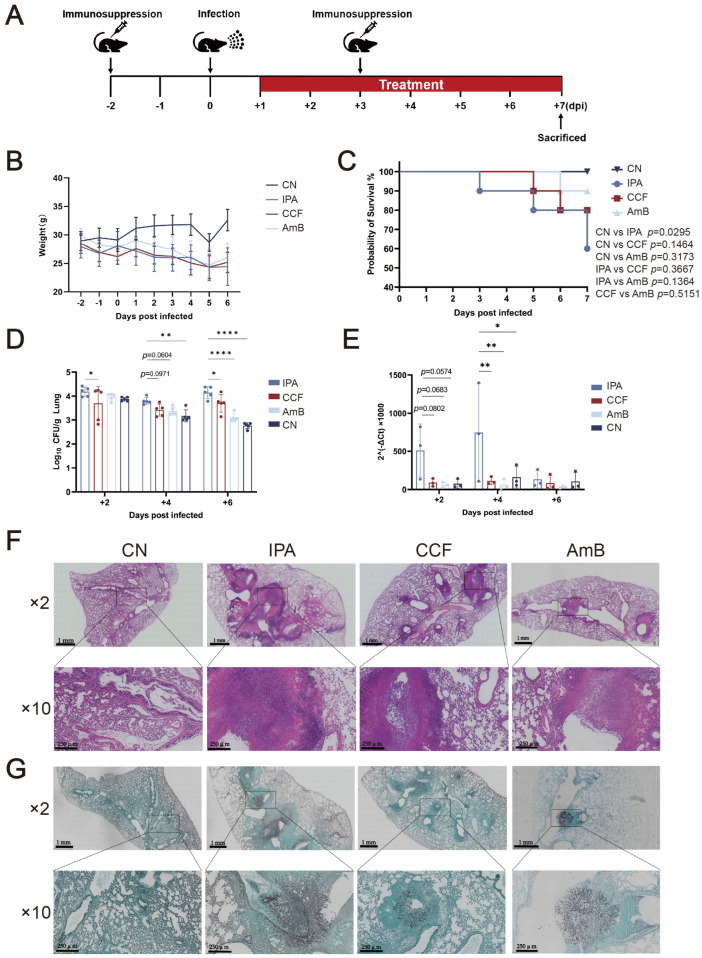
CCF improves survival and reduces pulmonary fungal burden and injury in IPA mice, with efficacy comparable to AmB. (**A**) Experimental schedule of IPA model establishment and treatment. Immunosuppression was induced at −2 and +3 dpi. Mice were infected intranasally with *A. fumigatus* AF293 spores at +0 dpi. CCF and AmB treatments were administered starting at +1 dpi. (**B**) Body weight changes in each group during the experiment. Data are presented as mean ± SD from *n* = 10 mice per group. (**C**) Survival curves of mice in each group up to +7 dpi. *n* = 10 mice per group. (**D**) Fungal burden in lung tissues assessed by colony-forming unit (CFU) counts at +2, +4, and +6 dpi. Statistical analysis was conducted using one-way ANOVA, comparing all groups to the IPA group. * *p* < 0.05, ** *p* < 0.01, **** *p* < 0.0001 indicate levels of statistical significance. Data are presented as mean ± SD from *n* = 5 mice per group. (**E**) Fungal load determined by qPCR analysis of *A. fumigatus* 18S rDNA expression in lung tissues at +2, +4, and +6 dpi. Statistical analysis was conducted using one-way ANOVA, comparing all groups to the IPA group. * *p* < 0.05, ** *p* < 0.01 indicate levels of statistical significance. Data are presented as mean ± SD from *n* = 3 mice per group. (**F**) Representative histopathological changes in lungs at +6 dpi shown by H&E staining. (**G**) Representative histopathological changes in lungs at +6 dpi shown by GMS staining.

To evaluate the therapeutic efficacy of CCF against IPA, *A. fumigatus* AF293 was used to establish an infection model, with immunosuppression induced at −2 days and +3 days post-infection (dpi). At +1 dpi, mice were divided into four groups (*n* = 10 per group): CN group (immunocompetent infected control; infected without immunosuppression), IPA group (immunodeficient with infection), CCF group (immunodeficient with infection and CCF treatment), and AmB group (immunodeficient with infection and AmB treatment) (Figure 1A). Body weight and survival were monitored daily until +7 dpi. Following infection, both CCF and AmB groups showed faster weight recovery compared to the IPA group (Figure 1B). By +7 dpi, survival declined to 60% in the IPA group, whereas AmB maintained survival above 90%; CCF treatment resulted in 90% survival at +5 dpi, slightly decreasing to 80% by +7 dpi (Figure 1C). Fungal burden analysis showed a time-dependent reduction in CFU counts and 18S rDNA expression in the CCF group, with significant differences observed at +2 dpi and +6 dpi for CFU (*p* < 0.05) (Figure 1D) and at +4 dpi for 18S rDNA (*p* < 0.01) (Figure 1E), compared with the IPA group. Histopathological examination at +6 dpi revealed extensive abscess formation, dense hyphal invasion, and severe inflammatory infiltration in the IPA group, whereas CCF and AmB treatments led to reduced fungal burden, fragmented hyphae, and less pronounced tissue edema (Figure 1F,G). Collectively, CCF demonstrated potent efficacy in improving survival, clearing fungal burden, and mitigating lung injury, approaching the performance of AmB under these experimental conditions and positioning it as a promising therapeutic candidate for IPA.

### 3.2. CCF Has No Hepatorenal Toxicity in IPA Mice

**Figure 2:**
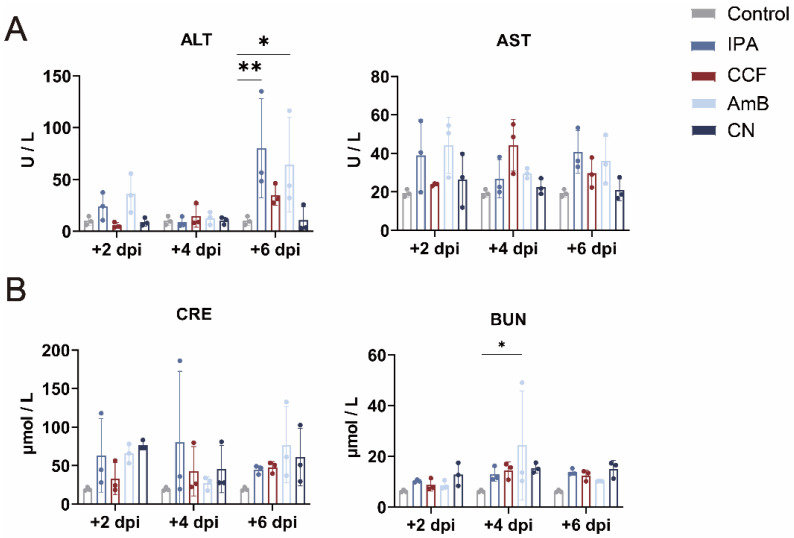
CCF shows no evident hepatorenal toxicity, with liver and kidney indices remaining near baseline relative to IPA/AmB. (**A**) Changes in serum ALT and AST levels in each group at +2, +4, and +6 dpi. Statistical analysis was conducted using one-way ANOVA, comparing all groups to the Control group. * *p* < 0.05, ** *p* < 0.01 indicate levels of statistical significance. Data are presented as mean ± SD from *n* = 3 mice per group. (**B**) Changes in serum BUN and CRE levels in each group at +2, +4, and +6 dpi. Statistical analysis was conducted using one-way ANOVA, comparing all groups to the Control group. * *p* < 0.05, indicate levels of statistical significance. Data are presented as mean ± SD from *n* = 3 mice per group.

To assess the potential hepatorenal toxicity of CCF in IPA treatment, we measured liver (ALT and AST) and kidney (BUN and CRE) function biomarkers. The naïve control group (uninfected, untreated), consisting of age-matched mice without immunosuppression, infection, or drug administration, served as the baseline reference. At +6 dpi, ALT levels in the IPA and AmB groups were significantly higher than in the Control groups (*p* < 0.05 and *p* < 0.001), indicating liver damage (Figure 2A). In contrast, the CCF group showed no significant differences in ALT and AST compared to the Control group, suggesting no hepatotoxicity. Kidney function was assessed by measuring BUN and CRE. The AmB group showed elevated BUN at +4 dpi (*p* < 0.05), whereas BUN and CRE levels in the CCF group remained similar to those in the control group, indicating no nephrotoxic effects (Figure 2B). These findings establish the superior safety profile of CCF, delivering antifungal efficacy without compromising hepatic or renal function. This may represent a meaningful advantage over conventional AmB therapy.

### 3.3. CCF Protects IPA Mice by Modulating the Immune System

**Figure 3:**
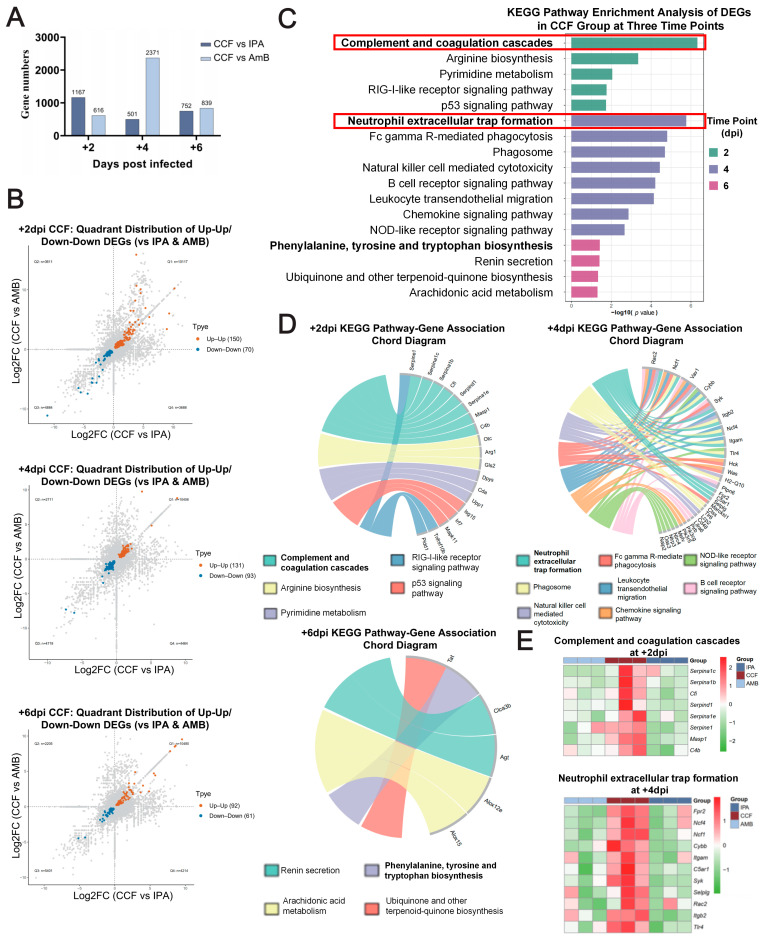
CCF-specific Up-Up genes show a temporal transcriptional program consistent with staged immunomodulation. Complement-related signals are most prominent at +2 dpi, whereas NET-related pathway enrichment becomes more evident at +4 dpi. (**A**) Numbers of differentially expressed genes from pairwise comparisons at +2, +4, and +6 days post infection: IPA vs. CCF (dark blue) and CCF vs. AmB (light blue). (**B**) Scatterplots of log2 fold change (log2FC) in CCF vs. IPA (x-axis) against CCF vs. AmB (y-axis) at each time point. Genes showing the same direction of change in both comparisons are highlighted as CCF-specific DEGs: Up-Up (orange) and Down-Down (blue). (**C**) KEGG pathway enrichment of CCF-specific DEGs at +2, +4, and +6 dpi, ranked by −log10 (*p* value); bar colors denote time points. Red boxes highlight the most significantly enriched KEGG pathways at +2 dpi and +4 dpi. (**D**) Chord diagrams mapping representative significantly enriched pathways to their constituent genes at each time point; ribbons connect pathways to member genes, with width indicating contribution within the pathway. (**E**) Heatmaps of representative pathways: “Complement and coagulation cascades” at +2 dpi and “Neutrophil extracellular trap formation” at +4 dpi. Expression values are scaled per gene (row-centered and Z-scored); color encodes relative expression across IPA, CCF, and AmB groups.

To further investigate the mechanisms underlying the effects of CCF against IPA, we performed lung RNA-seq at +2, +4, and +6 dpi in IPA, CCF, and AmB groups (Figure 3A), yielding 1167/616, 2371/501, and 752/839 differentially expressed genes (DEGs) for CCF vs. IPA/CCF vs. AmB, respectively. Using a concordant-direction filter, we defined Up-Up genes as CCF-specific DEGs, totaling 150, 131, and 62 at +2, +4, and +6 dpi (Figure 3B). KEGG analysis of these CCF-specific DEGs revealed stage-specific programs (Figure 3C,D). At +2 dpi, the enriched pathways mainly involved complement and coagulation cascades, arginine and pyrimidine metabolism, and RIG-I-like receptor and p53 signaling. At +4 dpi, the dominant pathways shifted to neutrophil extracellular trap formation, Fc gamma R-mediated phagocytosis, phagosome, natural killer cell-mediated cytotoxicity, and leukocyte transendothelial migration/chemokine signaling. At +6 dpi, the enriched pathways were mainly related to phenylalanine, tyrosine, tryptophan, and arachidonic acid metabolism, ubiquinone biosynthesis, and renin secretion. Heatmaps (Figure 3E) show the top pathway at each time point: complement and coagulation cascades at +2 dpi, with *C4b*, *Cfi*, and *Masp1* elevated in the CCF group, and neutrophil extracellular trap formation at +4 dpi, with Itgam, *Ncf1*, *Ncf4*, *Itgb2*, *Cybb*, and *Rac2* upregulated in CCF versus IPA and AmB. Together, these data suggest that CCF is associated with time-dependent immune transcriptional changes in IPA mice.

### 3.4. WGCNA Reveals a +2dpi-Specific Module Enriched with Complement-Related Transcriptional Signatures

**Figure 4:**
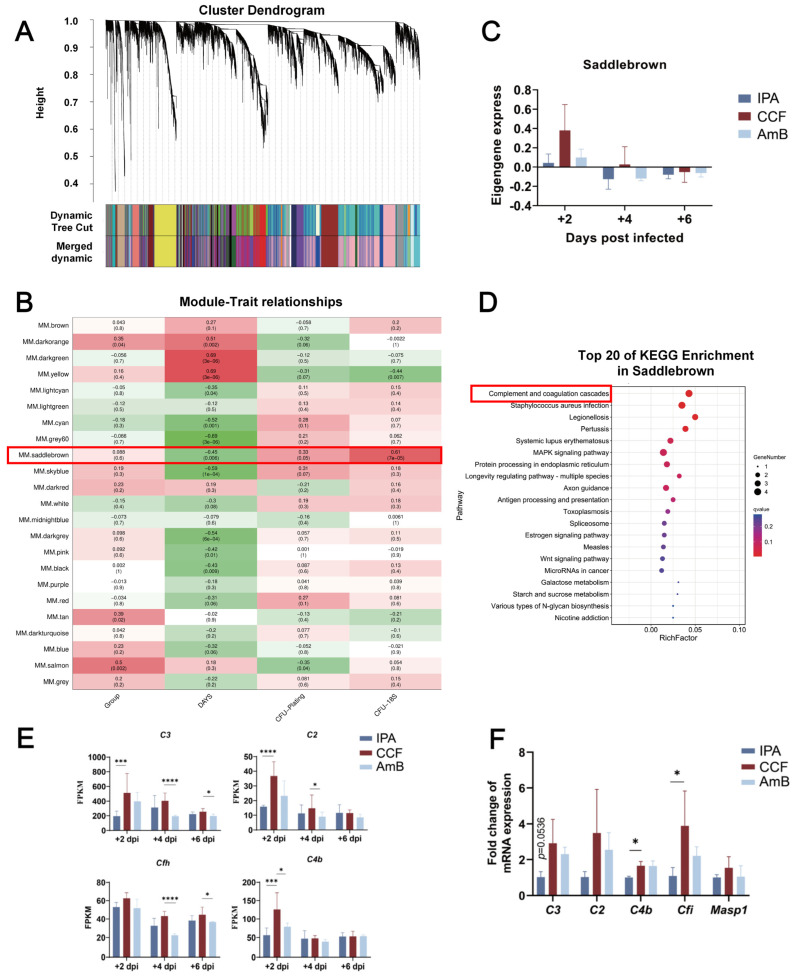
WGCNA identifies a +2 dpi module enriched for complement genes that is upregulated by CCF. The key feature of this figure is the Saddlebrown module, which links the early CCF-associated response to complement and coagulation cascade genes. (**A**) Hierarchical clustering tree showing the module structure based on RNA-Seq data. Different colors represent distinct clusters identified by the analysis (**B**) Correlation analysis between module eigengenes and experimental traits, including group, time point, CFU, and *A. fumigatus* 18S rDNA expression. Red denotes positive correlation, green denotes negative correlation. Color intensity reflects correlation strength. The red box highlights the Saddlebrown module. (**C**) Expression pattern of the Saddlebrown module eigengenes in each group at +2, +4, and +6 dpi. (**D**) KEGG pathway enrichment of genes within the Saddlebrown module. The red box highlights the most significantly enriched KEGG pathway. (**E**) FPKM values of genes from the “complement and coagulation cascade” pathway within the Saddlebrown module at +2, +4, and +6 dpi. Statistical analysis was conducted comparing all groups to the CCF group. * *p* < 0.05, *** *p* < 0.001, **** *p* < 0.0001 indicate levels of statistical significance. (**F**) qPCR validation of complement-related genes expression at +2 dpi. Statistical analysis was conducted using one-way ANOVA, comparing all groups to the CCF group. * *p* < 0.05 indicate levels of statistical significance. Data are presented as mean ± SD from *n* = 5 mice per group.

To elucidate the regulatory mechanisms of CCF in IPA, we performed WGCNA using RNA-Seq data. A total of 13,539 genes were clustered into 20 modules (Figure 4A), among which 10 were significantly associated with treatment groups (*p* < 0.05), time points, CFU counts and *A. fumigatus* 18S rDNA expression (Figure 4B). The Saddlebrown module exhibited the correlation with time points and was upregulated by CCF at +2 dpi (Figure 4C). KEGG pathway enrichment identified the “complement and coagulation cascade” as the most enriched pathway in this module (Figure 4D), comprising *C3*, *C2*, *Cfh*, and *C4b*, with expression analysis confirming their significant upregulation at +2 dpi (Figure 4E). To further validate these observations, qPCR was performed to assess the mRNA expression levels of *C3*, *C2*, *C4b*, together with CCF-specific expressing gene *Cfi*, and *Masp1* at +2 dpi (Table S3-1 and Figure S2). Compared with the IPA group, CCF treatment significantly upregulated *C4b* and *Cfi* expression, while *C3* showed an increasing trend (Figure 4F). These findings suggest that CCF confers protection against IPA by transcriptionally upregulating complement-related genes at +2 dpi, consistent with early engagement of complement pathways, while functional complement activation remains to be directly validated.

### 3.5. Bayesian Network Analysis Identifies Key Hub Genes in the CCF Group Linked to NADPH Oxidase Signaling

**Figure 5:**
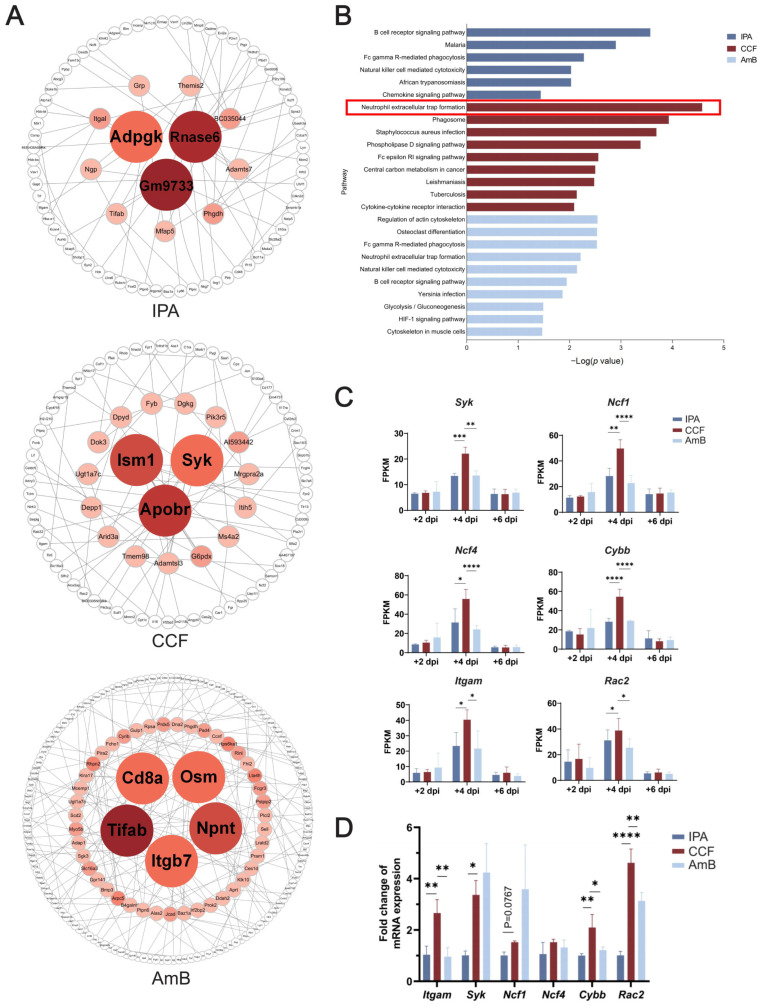
Bayesian networks and KEGG suggest a CCF-biased reorganization toward CR3-SYK-NOX/NET pathways relative to IPA/AmB. The network analysis highlights recurrent CCF-associated hub genes, including Itgam, Syk, Rac2, and Cybb, together with pathway enrichment linked to oxidative and NET-related responses. (**A**) Hub gene networks identified in the IPA, CCF, and AmB groups by Bayesian network analysis. Genes were ranked by DEGREE value, with the top 10% defined as hub genes and marked in color; nodes with DEGREE >6 were placed at the core of the network. (**B**) KEGG pathway enrichment of hub genes in each group. Pathways were ranked by −log_10_ (*p* value). Bar colors represent different groups: IPA (dark blue), CCF (dark red), and AmB (light blue). The red box denotes the most significantly enriched KEGG pathway in the CCF group. (**C**) FPKM values of key CCF-regulated genes at +2, +4, and +6 dpi. Statistical analysis was conducted comparing all groups to the CCF group. * *p* < 0.05, ** *p* < 0.01, *** *p* < 0.001, **** *p* < 0.0001 indicate levels of statistical significance. (**D**) qPCR validation of key CCF-regulated genes in each group. Statistical analysis was conducted using one-way ANOVA, comparing all groups to the CCF group. * *p* < 0.05, ** *p* < 0.01, **** *p* < 0.0001 indicate levels of statistical significance. Data are presented as mean ± SD from *n* = 3 mice per group.

To identify core regulatory genes within the immune network modulated by CCF in IPA, we performed Bayesian network analysis based on time course RNA-Seq data from the IPA, CCF, and AmB groups. The CCF group exhibited a unique hub gene profile, with *Apobr* (DEGREE = 8), *Ism1* (DEGREE = 8), and *Syk* (DEGREE = 6) being highly connected within immune regulatory networks (Figure 5A). In contrast, the IPA group showed a dysregulated immune response, with hub genes such as *Gm9733* (DEGREE = 11), *Rnase6* (DEGREE = 10), and *Adpgk* (DEGREE = 6) primarily associated with metabolic processes and extracellular matrix remodeling, reflecting an immune-compromised state (Figure 5A). Meanwhile, the AmB group exhibited a distinct hub gene profile, with *Tifab* (DEGREE = 11), *Npnt* (DEGREE = 8), *Osm* (DEGREE = 6), *Cd8a* (DEGREE = 6) and *Itgb7* (DEGREE = 6) linked to immune modulation and metabolic adaptation (Figure 5A).

KEGG pathway enrichment analysis revealed significant enrichment of the neutrophil extracellular trap formation pathway and phagosome pathway in the CCF group, consistent with enhanced fungal clearance capacity (Figure 5B). In the IPA group, enrichment in B cell receptor signaling, malaria, and Fc gamma R-mediated phagocytosis indicated a compromised yet ongoing immune reaction. In contrast, the AmB group showed enrichment in regulation of actin cytoskeleton and Fc gamma R-mediated phagocytosis, suggesting that AmB may promote fungal elimination and tissue repair through modulation of immune signaling and cytoskeletal dynamics.

NET formation pathways were specifically enriched in the CCF group at +4 dpi, as shown in the previous results (Figure 5D). The constituent genes identified in this pathway include *Itgam*, *Selplg*, *Ncf1*, *Syk*, *C5ar1*, *Ncf4*, *Itgb2*, *Rac2*, *Cybb*, *Fpr2*, *Tlr4*, and *Plcb1* (Table S3-2). In comparison, the KEGG analysis from the Bayesian network identified a slightly different set of genes involved in the NET formation pathway, including *Itgam, Selplg*, *Syk*, *Ncf2, Fcgr4*, *Fpr1*, *Rac2*, *Fpr2*, and *Plcb1*. Comparing these two NET-related pathway outputs, we identified the constituent genes of these pathways and identified six key genes-*Itgam*, *Syk*, *Ncf1*, *Ncf4*, *Rac2*, and *Cybb*-that were recurrent across multiple immune pathways and specifically upregulated in the CCF group (Figure 5C and Table S3-2). Notably, *Ncf1*, *Ncf4*, *Rac2*, and *Cybb* are core components of the NADPH oxidase complex, implicating this enzyme system in CCF-mediated enhancement of ROS-dependent antifungal responses. qPCR validation confirmed the upregulation of *Itgam*, *Syk*, *Cybb*, and *Rac2* in the CCF group, supporting their functional relevance in host defence (Figure 5D).

Together, these findings indicate that CCF is associated with enrichment of NET-related transcriptional programs and may exert part of its protective effect through SYK-linked oxidative host responses, which are consistent with enhanced antifungal activity against *A. fumigatus*.

### 3.6. CCF Confers Protection Against IPA with Evidence Consistent with Oxidative Pathway Involvement

**Figure 6:**
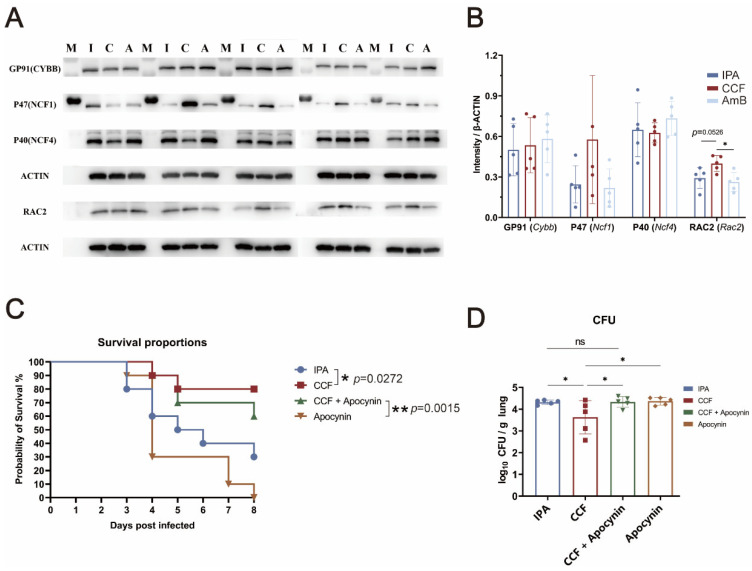
CCF enhances NOX2 components and its benefit is partially attenuated by apocynin, consistent with oxidative pathway involvement. This figure combines protein, survival, and fungal-burden data to show partial pharmacological support for the role of oxidative responses in CCF-mediated protection. (**A**) Protein expression of NADPH oxidase subunits in lung tissue at +4 dpi as detected by Western blot. I, IPA group; C, CCF group; A, AmB group; M, Marker. Data are presented as mean ± SD from *n* = 5 mice per group. (**B**) Densitometric quantification of protein levels normalized to β-ACTIN. Statistical analysis was performed using one-way ANOVA with multiple comparisons. * *p* < 0.05. Data are presented as mean ± SD from *n* = 5 mice per group. (**C**) Survival curves of mice up to +8 dpi. CCF significantly improved survival compared to the IPA group. Co-treatment with the NADPH oxidase inhibitor apocynin partially reversed this effect. * *p* < 0.05, ** *p* < 0.01. Survival data were analyzed using the Kaplan–Meier method with *n* = 10 mice per group. (**D**) Fungal burden in lung tissues at +4 dpi measured by colony-forming unit counts. Statistical analysis was performed using one-way ANOVA with multiple comparisons. * *p* < 0.05. Data are presented as mean ± SD from *n* = 5 mice per group.

To explore whether oxidative pathways contribute to the protective effect of CCF during *A. fumigatus* infection, we first assessed the protein levels of key NADPH oxidase-related subunits at +4 dpi by Western blot. Compared with the AmB group, the CCF group showed markedly higher RAC2 expression, a core component of the NADPH oxidase complex, while modest increases were also observed in NCF1 (Figure 6A,B).

To further examine whether oxidative pathways contribute to CCF-mediated protection, apocynin was co-administered with CCF. Previous studies have shown that apocynin can interfere with oxidase assembly by limiting the membrane translocation of cytosolic subunits such as p47phox and p67phox [21]. At +8 dpi, survival in the CCF group reached 80%, significantly higher than the IPA group (30%) (Figure 6C). CCF + Apocynin partially restored survival (60%) compared to Apocynin alone (0%, *p* = 0.0015). CFU analysis at +4 dpi showed significantly lower fungal burden in the CCF group than in the IPA, CCF + apocynin, and apocynin groups (Figure 6D, *p* = 0.0466). In contrast, neither CCF + Apocynin nor Apocynin alone showed significant differences from the IPA group. CCF improved survival and reduced fungal burden, and these benefits were partly attenuated by apocynin. Together, these results support the contribution of oxidative (NADPH oxidase/ROS) pathways to CCF-mediated protection, while pharmacological inhibition alone does not prove pathway-specific dependence.

### 3.7. Impact of CCF on Gut Microbiota Composition and Its Correlation with NADPH Oxidase Genes

**Figure 7:**
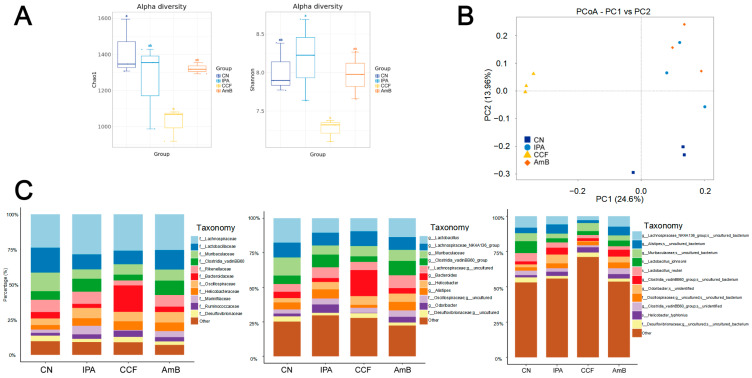

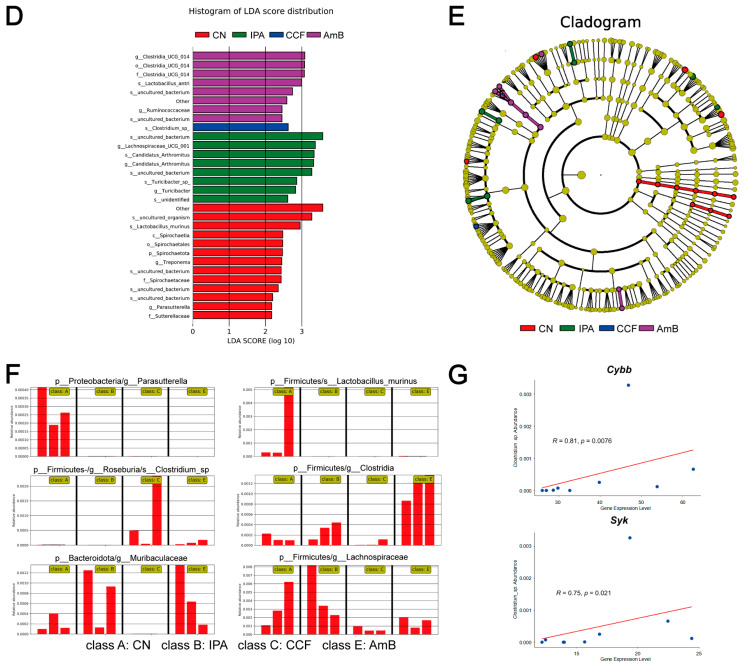
CCF-treated mice show microbiota remodeling with enrichment of *Clostridium* sp., and observed *Cybb/Syk* associations are non-causal. The main features of this figure are the distinct clustering of gut microbial communities across groups, the selective enrichment of *Clostridium* sp. in the CCF group, and its positive correlation with the redox-related host genes *Cybb* and *Syk*. (**A**) Alpha diversity analysis using the Chao1 index and Shannon index reveals significant differences among CN, IPA, CCF, and AmB groups. (**B**) Principal coordinates analysis (PCoA) based on weighted UniFrac distances demonstrates distinct clustering of gut microbial communities across groups. (**C**) Taxonomic composition profiles at the family genus, and species levels. Bar plots display the relative abundance of dominant taxa within each group. (**D**) Histogram of LDA scores (log_10_) for discriminative features, indicating significantly enriched taxa in each group. The colors represent different groups: CN (blue), IPA (red), CCF (green), and AmB (purple). (**E**) Cladogram representing the phylogenetic distribution of differentially abundant taxa among the groups. Colored nodes represent taxa significantly enriched in the corresponding groups: CN (blue), IPA (red), CCF (green), and AmB (purple). From inner to outer circles represent phylum, class, order, family, genus, and species levels. Taxa connected by the same color belong to the same species. (**F**) Comparison of the abundances of significantly different biomarkers: g__*Parasutterella* and s__*Lactobacillus* in the CN group, s__*Clostridium_sp* in the CCF group, g__*Clostridia* in the AmB group, and significantly deleted biomarkers g__*Muribaculaceae* and g__*Lachnospiraceae* in the CCF group. (**G**) Spearman correlation analysis between *Clostridium* sp. abundance and the expression levels of NADPH oxidase-related genes. Scatter plots show strong positive correlations with *Cybb* (R = 0.81, *p* = 0.0076) and *Syk* (R = 0.75, *p* = 0.021).

To explore the role of gut microbiota in the systemic effects of CCF, we conducted full-length 16S rDNA sequencing of fecal samples. Alpha diversity analysis using the Chao1 and Shannon indices revealed significant differences among the CN, IPA, CCF, and AmB groups. Specifically, both microbial richness (Chao1 index) and diversity (Shannon index) were significantly lower in the CCF group compared to both the IPA and AmB groups, suggesting a reduction in microbial diversity in the CCF-treated animals (Figure 7A). Despite this reduction, PCoA showed clear separation of microbial profiles in the CCF group (Figure 7B), and taxonomic bar plots demonstrated marked compositional differences at the family, genus, and species levels (Figure 7C). LEfSe analysis identified discriminative taxa across the groups, with s_*Clostridium* sp. enriched in the CCF group, and g_*Lachnospiraceae* and g_*Clostridia* predominant in the IPA and AmB groups, respectively. The CN group showed significant enrichment of *Parasutterella* and s__*Lactobacillus murinus* (Figure 7D). These results were further confirmed by LDA score histograms, cladogram analysis, and relative abundance plots (Figure 7E). LEfSe analysis further identified distinct biomarkers across groups. Specifically, g_*Parasutterella* and s__*Lactobacillus murinus* were significantly enriched in the CN group. In the CCF group, s_*Clostridium* sp. was significantly enriched, while *Clostridia* was notably enriched in the AmB group. Additionally, g__*Muribaculaceae* and g__*Lachnospiraceae* were significantly depleted in the CCF group (Figure 7F). Spearman correlation analysis revealed that the abundance of *Clostridium* sp. was significantly positively correlated with the expression levels of *Cybb* (R = 0.81, *p* = 0.0076) and *Syk* (R = 0.75, *p* = 0.021), suggesting a potential link between this taxon and NADPH oxidase-mediated immune responses (Figure 7G; other genes in Figure S3). Together, these findings identify *Clostridium* sp. as a taxon enriched in the CCF group and positively correlated with the NADPH oxidase-related genes *Cybb* and *Syk*. Along with the upregulation of pulmonary NADPH oxidase components, this pattern suggests an exploratory link between microbiota remodeling and redox-related immune signatures along the gut–lung axis.

## 4. Discussion

IPA is a severe opportunistic fungal infection that primarily affects immunocompromised individuals. Its progression is often accompanied by excessive inflammatory responses and poor immune clearance, leading to high mortality in clinical practice [22]. Conventional antifungal drugs, while available, are frequently associated with limited efficacy due to dose-related toxicity and resistance [23]. CCF, a well-known traditional Chinese medicinal herb, is widely used in inflammatory disorders and has been reported to reduce systemic inflammation and restore immune balance in several disease models. Key alkaloids derived from CCF, such as sanguinarine and chelerythrine, have demonstrated anti-inflammatory and analgesic effects without causing gastrointestinal toxicity [24]. Water extracts of CCF have also been shown to mitigate sepsis severity in mice by modulating gut microbiota and metabolic profiles, thereby maintaining immune homeostasis [16]. In this study, we aimed to investigate the therapeutic potential of CCF in treating IPA and to provide insights that may assist future clinical strategies. We focused on its immunomodulatory functions and the possible involvement of gut microbiota–redox signaling axes in mediating antifungal protection.

In our murine IPA model, CCF showed clear therapeutic efficacy in vivo and may offer useful insight for future host-directed treatment strategies. Survival analysis revealed that CCF treatment significantly improved survival, with survival rates of 90% at +5 dpi and 80% at +7 dpi, compared to 60% in the IPA group. These outcomes closely mirrored those of the AmB group, where survival remained above 90%. The improved survival rate was accompanied by a marked reduction in fungal burden, as reflected by lower CFU counts in lung tissues at +2 and +6 dpi in the CCF-treated group compared to the IPA group. While this reduction is often interpreted as a sign of enhanced immune clearance, alternative or complementary mechanisms may also contribute. In particular, it is plausible that CCF limits fungal establishment in the lung by interfering with *A. fumigatus* adhesion or invasion processes, potentially contributing to reduced fungal burden alongside immune mechanisms, even in the absence of direct evidence for overt immune activation. Berberine, the principal alkaloid of CCF, has been shown to significantly inhibit Candida albicans adhesion to epithelial cells by downregulating ICAM-1, MUC1, and MUC4 expression [25]. Such interference with pathogen–host interactions may provide a mechanistic explanation for the reduced fungal burden observed in our model. In addition to reduced CFU, 18S rDNA expression, a molecular indicator of total fungal biomass, was also significantly decreased following CCF treatment at +4 dpi. This suggests that the observed fungal clearance may result not only from direct fungicidal activity, but also from enhanced innate immune responses. Berberine, one of the major isoquinoline alkaloids and pharmacologically active components of Coptis chinensis, has been shown to promote immune effector functions. Berberine has been shown to enhance inflammasome activation in murine macrophages via AMPK signaling, resulting in elevated IL-1β production and improved in vivo pathogen clearance [26]. Similarly, berberine hydrochloride has been reported to activate the p38 MAPK pathway, thereby enhancing innate immunity against bacterial infection [27]. Histopathological analysis further confirmed that CCF alleviated fungal colonization and inflammation in the lung, consistent with in vivo studies suggesting that coptisine, another active alkaloid from CCF, attenuates pulmonary tissue damage and fungal burden by disrupting fungal membranes and modulating oxidative stress pathways [28].

Hepatotoxicity and nephrotoxicity are major concerns with many antifungal treatments. However, in our study, CCF treatment did not cause liver or kidney damage, as shown by stable ALT, AST, BUN, and creatinine levels, which were comparable to the control group. In contrast, the IPA and AmB groups exhibited elevated ALT and AST levels, indicating liver damage, and BUN levels were significantly higher in the AmB group, suggesting nephrotoxicity. These findings are consistent with previous studies showing that AmB, while effective, frequently induces hepatic and renal toxicity in vivo [23]. The favorable hepatic and renal safety profile of CCF observed in our model aligns with earlier pharmacological studies reporting that berberine, the major alkaloid component of CCF, exerts antioxidative and cytoprotective effects under chemically induced organ stress. For example, berberine has been shown to significantly ameliorate acetaminophen-induced liver injury in vivo, as evidenced by normalized ALT and AST levels and reduced oxidative stress in a fish model [29]. While the cited study used a chemically induced injury model, the antioxidant and organ-protective effects of berberine observed therein are consistent with the lack of liver and kidney toxicity seen in our IPA model.

The therapeutic effects of CCF in IPA appear to be mediated through immune modulation. RNA-seq analysis revealed that CCF treatment significantly enriched complement- and coagulation-related genes at +2 dpi, consistent with early engagement of innate immune pathways at the transcriptional level. This finding is consistent with studies showing that activation of the complement system plays a crucial role in early immune defense against fungal infections [30]. By +4 dpi, genes associated with leukocyte transendothelial migration, chemokine signaling, and NET formation were upregulated, indicating enhanced immune cell recruitment to the infection site, with concordant upregulation of a NET/phagocytic module (*Itgam/Itgb2*, *Ncf1*, *Ncf4*, *Cybb*, *Rac2*) in CCF compared with IPA/AmB. This observation is consistent with mechanistic evidence showing that the loss of β2 integrin function in neutrophils significantly impairs their ability to migrate, generate reactive oxygen species, and form NETs, ultimately leading to elevated fungal burden and worsened outcomes in invasive pulmonary aspergillosis [31]. By +6 dpi, CCF shifted toward programs linked to inflammation resolution and homeostasis, emphasizing metabolic pathways (e.g., phenylalanine/tyrosine/tryptophan and arachidonic-acid metabolism, ubiquinone biosynthesis, and renin secretion), supporting a stage-resolved pattern whereby early innate activation is followed by amplified neutrophil effector functions and later metabolic reprogramming.

Using WGCNA, we identified a gene co-expression module (Saddlebrown) significantly enriched at +2 dpi following CCF treatment. This module contains key complement-related genes such as *C3*, *C2*, *C4b*, and *Cfh*, which were notably upregulated, implicating early complement cascade activation as a central feature of the CCF-mediated immune response. Its strong correlation with both fungal burden and infection time suggests that this module represents a crucial immune axis activated during the early phase of infection. This transcriptional signature is consistent with involvement of both classical and lectin complement pathways; however, functional complement activity (e.g., C3b/iC3b deposition or serum complement assays) was not directly measured in this study and remains to be validated. Supporting this, proteomic profiling has shown that *A. fumigatus* conidia bind directly to complement proteins including C3, C2, C4b, CFH, and MASP1 [32], thus promoting opsonization and neutrophil-mediated phagocytosis. However, their study also highlighted that bronchoalveolar lavage fluid (BALF) lacked C2 and MBL, suggesting that classical and lectin pathways predominate in serum, while the alternative pathway dominates in alveolar fluid, reflecting tissue-specific complement dynamics. The importance of C2 and C4b in complement activation was further confirmed in a mechanistic study showing that C2-deficient human serum failed to activate complement on *A. fumigatus* conidia, as evidenced by abolished C4b and C3b deposition, and that reconstitution with purified C2 restored complement activation [33]. This directly confirms the functional role of the classical/lectin pathway convertase (C4b2a) in initiating antifungal immune responses. Additionally, activation of the lectin pathway via MBL-MASP interaction has been described to lead to cleavage of C4 and C2, generating C4b2a [34]. Their review also reported that MBL supplementation enhances C4b deposition and fungal clearance in vitro and in vivo, further reinforcing the mechanistic link between lectin pathway activation and fungal recognition during infection. Complement-mediated protection is further supported in vivo by findings that C3-deficient mice exhibit significantly increased susceptibility to systemic *A. fumigatus* infection [35]. Interestingly, C4- and factor B-deficient mice did not display elevated fungal burden, indicating potential functional redundancy across complement pathways, with C3 acting as the central converging node [35]. Moreover, qPCR validation of CCF-treated lungs revealed upregulation of *Cfi* and *Masp1*, suggesting engagement of both regulatory (CFH/Cfi) and lectin-driven (MASP1) arms of complement during early fungal sensing and response. Together, these transcriptomic and functional data indicate that CCF rapidly mobilizes complement activation via multiple converging pathways, positioning complement as a frontline effector of antifungal defense.

Combined Bayesian network and KEGG analyses suggest that CCF is associated with dynamic enhancement of antifungal immunity, including transcriptional changes, immune network rewiring, and downstream ROS-related effector responses. In the CCF group, hub genes such as *Apobr*, *Ism1*, and *Syk* were highly connected within immune regulatory networks. Syk is a well-characterized immunoreceptor tyrosine kinase that plays a critical role in antifungal defense by mediating NADPH oxidase activation and ROS production in neutrophils, essential for the killing of fungal hyphae [36]. Ism1 has recently been shown to regulate lung immune homeostasis by promoting adiponectin expression and enhancing alveolar macrophage efferocytosis, thereby reducing airway inflammation and promoting resolution of immune responses [37]. Meanwhile, Apobr has been identified as an inflammation-associated gene, with expression elevated at sites of pathogen-induced tissue damage such as bacterial abscesses [38], and associated with host immune susceptibility in pediatric pulmonary infections [39], suggesting a potential role in leukocyte trafficking or lipid-mediated immune regulation during infection. In contrast, the IPA group exhibited a dysregulated immune architecture, with hub genes such as *Gm9733*, *Rnase6*, and *Adpgk* predominantly associated with metabolic processes and extracellular matrix (ECM) remodeling. This pattern indicates a deviation from immune-effector prioritization and reflects the immune-compromised state typical of uncontrolled fungal infections, where host defenses shift toward cellular stress responses rather than pathogen elimination [40]. Importantly, the AmB group, representing standard antifungal therapy, exhibited a distinct immune signature compared to CCF. While AmB was associated with activation of pathways such as Fc gamma receptor-mediated phagocytosis and cytoskeletal remodeling, it did not significantly enrich NET formation or NADPH oxidase activation pathways. This suggests that AmB may elicit a more limited oxidative immune response compared to the broad-spectrum immune activation observed with CCF.

Notably, two independent analyses are consistent with NET formation as a candidate antifungal mechanism preferentially associated with CCF treatment. First, KEGG enrichment of CCF-specific expressing genes at +4 dpi revealed that the NET formation pathway was significantly and exclusively enriched in the CCF group, with no comparable activation in IPA or AmB groups. Second, KEGG analysis of hub genes identified from Bayesian network inference also highlighted NET formation as a top-ranked pathway. Despite deriving from distinct analytical strategies, both approaches consistently identified a shared core gene module, *Itgam*, *Syk*, *Ncf1*, *Ncf4*, *Rac2*, and *Cybb*, which encode critical components of the NADPH oxidase complex, the central driver of ROS generation during neutrophil activation. These results align with prior mechanistic studies showing that fungal β-glucan recognition by the complement receptor 3 (CR3, also known as Itgam/Itgb2) initiates NET formation through activation of the SYK kinase and downstream NADPH oxidase-dependent ROS production [10]. In murine models of pulmonary aspergillosis, NADPH oxidase has been shown to be indispensable for NET formation and fungal clearance, with p47phox^−^/^−^ mice exhibiting a complete failure to form NETs and heightened fungal burden [41]. Similarly, it has been confirmed that disruption of NADPH oxidase severely impairs oxidative burst and NET-mediated immunity against *A. fumigatus*, while elastase deficiency alone has only partial effects [42]. Furthermore, NADPH oxidase activity in monocytes and macrophages has been shown to contribute to fungal clearance and regulation of lung inflammation, supporting a broader immune-regulatory function of this complex beyond neutrophils [43]. qPCR validation in our study confirmed the upregulation of *Cybb*, *Rac2*, *Itgam*, and *Syk*, supporting consistency with the activation of this signaling axis under CCF treatment. Although *Syk* expression in the AmB group was slightly higher than in the CCF group at the mRNA level, this difference was not statistically significant, suggesting that AmB may also induce partial SYK pathway activation. Nevertheless, the consistently higher expression of other NADPH oxidase components-*Ncf1*, *Ncf4*, *Cybb*, and *Rac2*-in the CCF group is consistent with a more robust and comprehensive engagement of the ROS-dependent antifungal mechanism by CCF. Taken together, these converging observations are consistent with the involvement of NET-related pathways and oxidative host responses associated with CR3-SYK-NADPH oxidase signaling. Interestingly, the earlier complement-related changes observed at +2 dpi may represent an upstream event associated with the later CR3-SYK-NADPH oxidase-linked response identified at +4 dpi. CR3 (CD11b/Itgam), which binds iC3b-opsonized fungi, not only showed transcriptional upregulation but is also functionally known to initiate intracellular signaling through SYK and activate NADPH oxidase-dependent NET formation [10]. This is consistent with a working model in which complement-related opsonization and CR3-associated signaling may help connect early pathogen sensing with later oxidative effector responses [44]. Taken together, these findings are compatible with coordinated involvement of the CR3-SYK-NADPH oxidase-NET axis and with ROS-dependent antifungal activity. This effector pathway appears deficient in the IPA group and only partially restored by AmB. Under our conditions, this integrated pattern was not evident with AmB.

To explore whether oxidative pathways contribute to the protective effect of CCF against *A. fumigatus*, we examined the expression of core NADPH oxidase-related proteins. Western blot analysis at +4 dpi revealed that CCF significantly upregulated RAC2 compared to the AmB group, with a modest increase in NCF1. Both RAC2 and NCF1 are essential cytosolic components of the phagocyte NADPH oxidase (NOX2) complex, which assembles at the membrane upon activation and catalyzes the production of ROS, a critical effector function of neutrophils during antifungal defense [43,45]. To confirm the functional relevance of NADPH oxidase activation, we co-administered the NADPH oxidase inhibitor Apocynin with CCF. Survival analysis revealed that CCF alone significantly improved survival (80%) compared to the IPA group (30%), whereas the survival benefit was partially attenuated by Apocynin (60%). Apocynin alone conferred no benefit (0%). Similarly, CFU analysis at +4 dpi showed that the CCF group had significantly lower fungal burden than IPA, CCF+ Apocynin, or Apocynin groups, with no significant differences among the latter three. These results provide partial support for the involvement of oxidative pathways in the protective efficacy of CCF and are consistent with enhanced oxidative antifungal responses. This is consistent with prior studies in both mammalian and zebrafish models, which demonstrate that NADPH oxidase-deficient hosts exhibit impaired fungal clearance, elevated fungal burden, and increased inflammatory pathology [43,46]. In addition, clinical evidence from patients with chronic granulomatous disease (CGD), caused by mutations in genes such as NCF1 or CYBB, reveals a profound susceptibility to invasive fungal infections, further supporting the critical role of the NADPH oxidase complex in host defense [47].

Given the emerging role of the gut–lung axis in shaping pulmonary immunity, we hypothesized that the antifungal effects of CCF might involve microbiota-derived signaling. Previous studies have shown that CCF modulates gut microbial composition and immune balance in systemic inflammatory settings. For example, CCF water extract has been reported to restore gut microbial diversity, increase anti-inflammatory metabolites, and regulate T cell responses in a sepsis model [16]. To explore whether CCF modulates antifungal immunity via gut microbiota-redox signaling, we performed 16S rDNA sequencing of fecal samples and identified significant alterations in microbial composition. At the phylum/class level, communities were dominated by *Firmicutes* (*Clostridia*) with group-specific shifts. Although alpha diversity was reduced in the CCF group, principal coordinate analysis showed distinct clustering, indicating a treatment-associated shift in gut microbial composition rather than a simple loss of diversity alone. A decrease in alpha diversity is often interpreted as a feature of gut microbiota dysbiosis [48]. However, reduced diversity has also been reported after berberine- or Coptis-based interventions, consistent with selective antimicrobial pressure and treatment-driven community reshaping [15]. Therefore, we interpret the CCF-associated microbiota changes as treatment-related remodeling rather than simple restoration, while acknowledging that their biological significance remains uncertain and requires functional validation. LEfSe analysis showed that *Clostridium* sp. was significantly enriched in the CCF group, in contrast to the dominance of *Lachnospiraceae* and *Clostridia* in IPA and AmB groups, respectively. Notably, *Clostridium* sp. abundance was positively correlated with the expression of *Cybb* and *Syk*, key components of the NADPH oxidase complex, suggesting a link between microbial changes and host redox signaling. This association is in line with previous findings showing that certain *Clostridium* species can influence host NADPH oxidase activity and ROS-related responses. For instance, *Clostridium difficile* Toxin B has been shown to activate a cytosolic NADPH oxidase-ROS-JNK-caspase-3 axis in enteric glial cells, bypassing mitochondrial stress and initiating microbiota-originated redox signaling [49]. Similarly, evidence has emphasized that gut-resident *Clostridium* and other anaerobes influence systemic inflammation via redox pathways, particularly involving NADPH oxidase-mediated ROS generation [50]. It has further been proposed that *Clostridium sporogenes* and related taxa participate in redox-sensitive immune training by enhancing neutrophil activation through NOX-dependent pathways [51]. In a broader context, Jones and Neish demonstrated that gut microbiota modulate host immunity and epithelial barrier function by engaging NOX1/2-dependent ROS signaling in response to microbial ligands such as formyl peptides [52]. Together, these findings support an exploratory model in which *Clostridium* sp. enrichment during CCF treatment is associated with redox-related host responses and antifungal immunity. These findings are consistent with a possible gut–lung association linking microbiota-derived signals to pulmonary neutrophil activation, although direct functional validation is still required. Accordingly, these microbiota-redox associations should be regarded as exploratory and non-causal until formal validation is available, for example, through antibiotic depletion or fecal microbiota transfer experiments.

Despite the robust evidence presented, several critical questions remain unresolved. First, while transcriptomic and functional data support the involvement of the CR3-SYK-NADPH oxidase-NET axis, the causal role of this pathway in mediating antifungal protection should be validated using targeted genetic models. For example, the use of CR3- or SYK-deficient mice could clarify the non-redundant role of upstream signaling components in NET formation and fungal clearance. Second, the gut–lung axis proposed in this study is based on correlational data linking *Clostridium* sp. abundance to the expression of NADPH oxidase-related genes. While prior studies support microbial redox signaling as a regulator of host immunity, direct mechanistic validation (e.g., through germ-free or microbiota transfer experiments) is required to confirm the functional role of gut bacteria in modulating pulmonary immune responses. Third, the multicomponent nature of CCF makes it challenging to determine which bioactive constituents are responsible for immune modulation. Future work involving bioactivity-guided fractionation and metabolomic profiling will be essential to identify key immunoregulatory compounds and facilitate their translational application. Moreover, a key temporal question remains unresolved: our data show complement activation (*C3*, *C2*, *C4b*) prominently upregulated at +2 dpi, whereas activation of the CR3-SYK-NADPH oxidase-NET axis is observed at +4 dpi. This raises the hypothesis that early complement-mediated opsonization may act as a priming event for subsequent CR3 engagement on neutrophils, triggering ROS-dependent NET formation. Testing this sequential immunological cascade using time-resolved blockade experiments or serum transfer models could provide new insight into the layered architecture of antifungal immunity.

While our study provides multi-faceted evidence supporting the role of CCF in enhancing antifungal immunity through the SYK-NADPH oxidase axis and a process consistent with NETs, we acknowledge several limitations as valuable directions for future investigation. First, direct morphological evidence of NET formation, such as immunofluorescence co-localization of MPO and citrullinated histone H3 (CitH3), was not obtained in the current study and remains to be established. Because the transcriptomic analysis was exploratory in nature, transcript-level findings should be interpreted as supportive of pathway-level inference rather than definitive evidence at the single-gene level. Furthermore, although transcriptional upregulation of NADPH oxidase components was observed, key post-translational events—particularly the phosphorylation of p47phox and its membrane translocation, essential for oxidase assembly—were not assessed at the protein level. Future studies using advanced imaging techniques will be needed to directly visualize NET structures, and further protein-level analyses may help define the phosphorylation dynamics underlying NADPH oxidase activation. Bioactivity-guided fractionation of CCF may help identify the specific alkaloids responsible for the observed immunomodulatory effects. In addition, a broader transcriptomic analysis incorporating the immunocompetent control group may further clarify the transition of host responses from health to infection and treatment, and may enable a more direct comparison between CCF and AmB.

**Figure 8:**
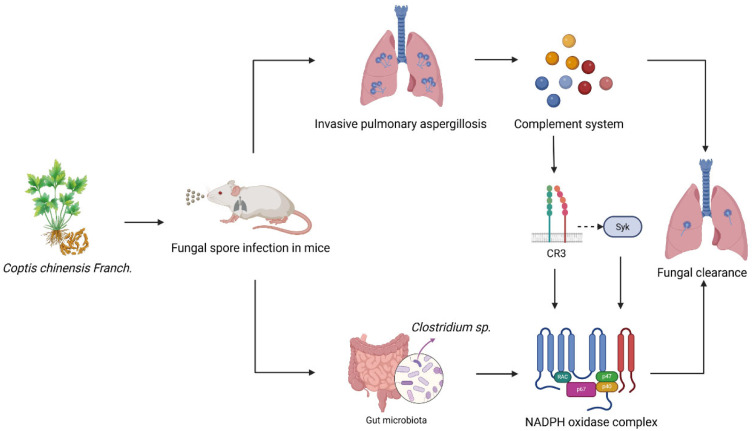
Proposed working model of the immune and microbiota changes associated with CCF treatment in invasive pulmonary aspergillosis. The schematic summarizes a staged response pattern, including early complement-related changes, later oxidative and neutrophil-related responses consistent with CR3-SYK-NADPH oxidase signaling and NET-related pathways, and concurrent gut microbiota remodeling. Together, these features provide an integrated, hypothesis-generating framework for interpreting the present findings. Created in BioRender. Jiang, Z. (2026) https://BioRender.com/l51kgw6 (accessed on 16 April 2026).

Taken together, our findings support a staged immune model associated with CCF treatment in invasive pulmonary aspergillosis, characterized by early complement-related changes, followed by oxidative and neutrophil-related responses, together with concurrent gut microbiota remodeling (Figure 8). This pattern provides a plausible framework for understanding how CCF may enhance antifungal host defense in vivo. However, this model should be regarded as hypothesis-generating rather than definitive, as the complement-related response was inferred mainly from transcriptomic analyses, the NET-related response was not directly visualized, and the microbiota association remains correlative. Overall, our data support CCF as a promising host-directed intervention in IPA and provide a basis for future mechanistic validation of the proposed model.

## 5. Conclusions

In summary, CCF enhanced survival, reduced fungal burden, and alleviated lung injury in a murine model of invasive pulmonary aspergillosis without apparent hepatotoxicity or nephrotoxicity. Time-resolved transcriptomic, pharmacological, and microbiota analyses demonstrated that its protective effects were linked to early complement-related responses, later redox- and neutrophil-related changes, and gut microbiota remodeling. These findings support the antifungal potential of CCF and provide a framework for further exploration of host-directed therapeutic strategies against IPA.

## Data Availability

The RNA-seq data and 16s rDNA data supporting the findings of this study have been deposited in the NCBI SRA repository under BioProject accession PRJNA1295008. The other data and Supplementary Materials that support the findings of this study are available from the corresponding author upon reasonable request.

## Supplementary Materials

The following supporting information can be downloaded at: https://www.mdpi.com/article/10.3390/cimb48040424/s1.
