# Supplementary material for: Coptis chinensis Franch. Suppresses Invasive Pulmonary Aspergillosis by Augmenting NADPH-Dependent Neutrophil Extracellular Traps via Dual Modulation of Complement Activation and Gut Microbiota
Source: Curr Issues Mol Biol. 2026 Apr 20;48(4):424. doi: 10.3390/cimb48040424 (PMC13115011; doi:10.3390/cimb48040424)
Supplement: Supplementary file 1 [file cimb-48-00424-s001.zip › cimb-4248685-Supplementary Materials.pdf]

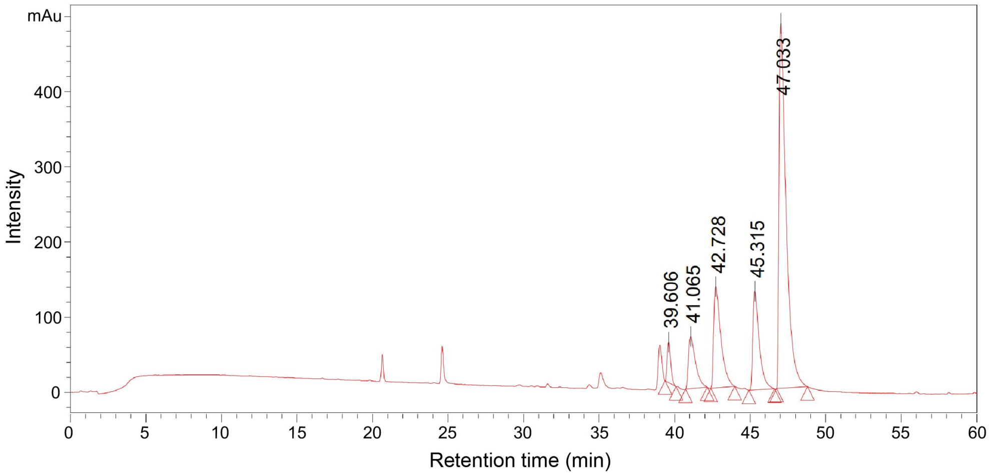

Figure S1

Chromatogram of CCF total alkaloids determined by HPLC. Peak identifications: jatrorrhizine (39.606 min), epiberberine (41.065 min), coptisine (42.728 min), palmatine (45.315 min), berberine (47.033 min).

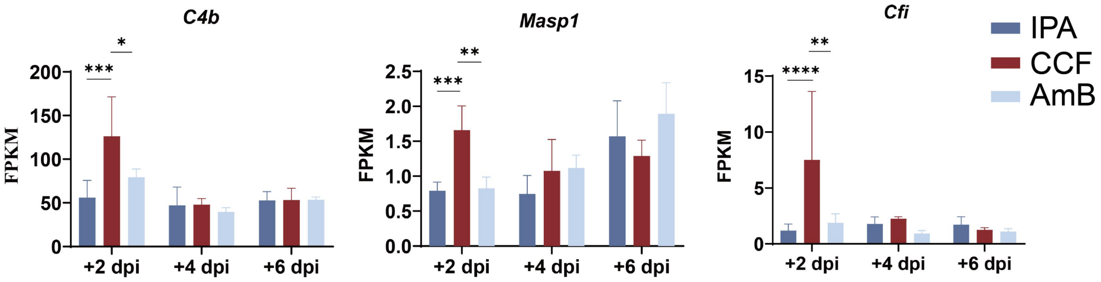

Figure S2

FPKM values of CCF-specific expressing gene in the "Complement and coagulation cascades" KEGG pathway at +2, +4, and +6 dpi. Statistical analysis was conducted comparing all groups to the CCF group. * P-value < 0.05, ** P-value < 0.01, *** P-value < 0.001， **** P-value < 0.0001 indicate levels of statistical significance.

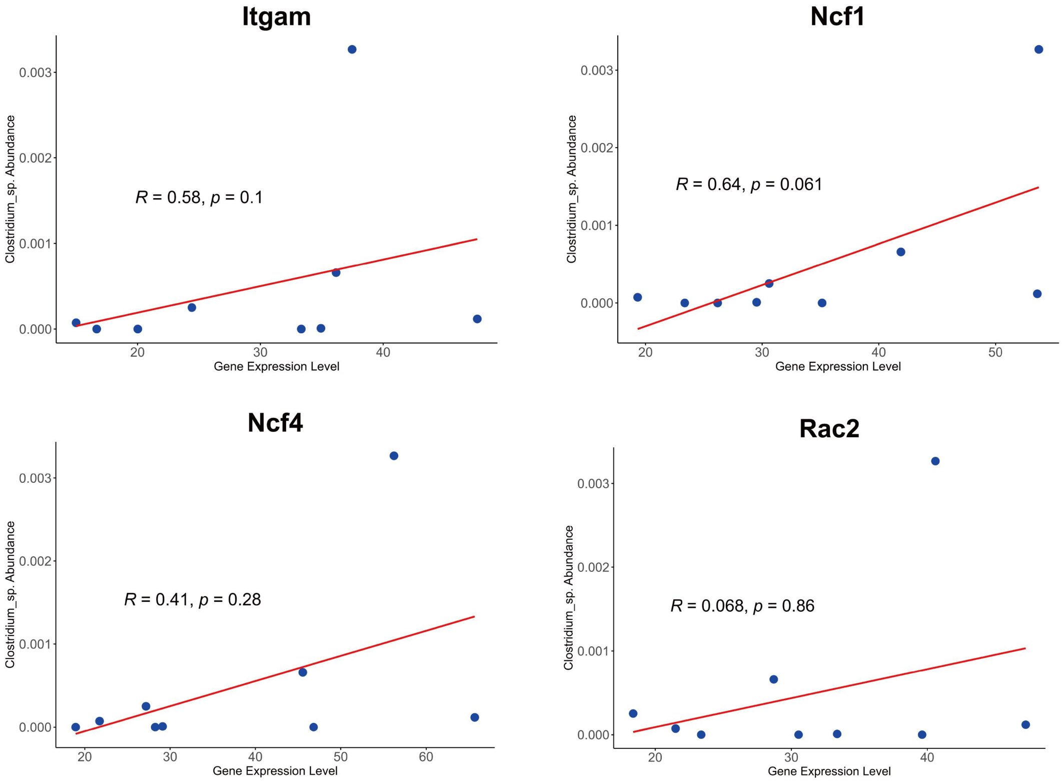

Figure S3

Spearman correlation analysis between *Clostridium sp*. abundance and the expression levels of NADPH oxidase-related genes. Scatter plots show strong positive correlations with *Itgam, Ncf1, Ncf4* and *Rac2*.

Table S1 CCF total alkaloids determined by HPLC

	Retention Time (min)	Peak Area(mAU·min)	Concentration(μ g/ml)
Jatrorrhizine	39.606	87741	122.348
Epiberberine	41.065	225008	286.661
Coptisine	42.728	447316	735.082
Palmatine	45.315	385619	391.334
Berberine	47.033	157899	2514.582

Table S2 Primer sequences used for qPCR analysis

gene	Forward (5'→3')	Reverse (5'→3')
Beta-Actin	AGCCATGTACGTAGCCATCC	CTCTCAGCTGTGGTGGTGAA
AF293 18S	ATGGCCGTTCTTAGTTGGTG	GAGCCGATAGTCCCCCTAAG
C3	CCAGCTCCCCATTAGCTCTG	GCACTTGCCTCTTTAGGAAGTC
C2	AGGTCTAGCTCACATCACACC	GCCGAGGGAAATAGATGCCATT
C4b	AAACTGTCCCCTGAAACAAAGG	AGTGCCACTGTCTCGTCAGA
Cfi	TCTTGGCTCTCCACTTGAGTT	GGAGCGATGCGTGTATTTCTG
Masp1	GACAGCCACAAGGTGACCATCA	GAAATGCTGCCATTAGGTGGCG
Itgam	CCATGACCTTCCAAGAGAATGC	ACCGGCTTGTGCTGTAGTC
Syk	GAGAGCACTGTGTCCTTCAACC	CAGCATAAGGGCTCTCGTACAC
Ncf1	GCTGACTACGAGAAGAGTTCGG	CCTCGCTTTGTCTTCATCTGGC
Ncf4	CAAAGACCTGCTAGCGCTCATG	CCACATCCTCATCTGACAGCAG
Cybb	TGGCGATCTCAGCAAAAGGTGG	GTACTGTCCCACCTCCATCTTG
Rac2	GACAGTAAGCCGGTGAACCTG	CTGACTAGCGAGAAGCAGATG

Table S3-1 KEGG pathway enrichment analysis of CCF-specific DEGs at +2 dpi

Term	Count	P Value	Genes
Complement and coagulation cascades	8	1.20×10^{-4}	*C4b, Cfi, Masp1, Serpina1b, Serpina1c, Serpind1, Serpine1, Serpina1e*
ECM-receptor interaction	7	6.00×10^{-4}	*Comp, Col2a1, Col4a1, Tnc, Col9a1, Col9a2, Thbs2*
PI3K-Akt signaling pathway	12	0.003471	*Chrm2, Comp, Pdgfra, Col2a1, Angpt1, Col4a1, Tnc, Col9a1, Col9a2, Thbs2, Prlr, Pdk1*

Table S3-2 KEGG pathway enrichment analysis of CCF-specific DEGs at +4 dpi

Term	Count	PValue	Genes
Leukocyte transendothelial migration	10	2.94×10^{-5}	*Cldn5*, ***Itgam***, *Cxcl12*, ***Ncf1***, ***Ncf4***, *Itgb2*, ***Rac2***, ***Cybb***, *Esam*, *Vav1*
Neutrophil extracellular trap formation	12	1.14×10^{-4}	***Itgam***, *Selplg*, ***Ncf1***, ***Syk***, *C5ar1*, *Ncf4*, *Itgb2*, ***Rac2***, ***Cybb***, *Fpr2*, *Plcb1*, *Tlr4*
Phagosome	10	7.89×10^{-4}	***Itgam***, ***Ncf1***, ***Ncf4***, *Itgb2*, *Cybb*, *Tlr6*, *Tlr4*, *H2-Q1*, *Ctss*, *H2-Q10*
Fc gamma R-mediated phagocytosis	7	0.00159	*Hck*, *Marcksl1*, ***Ncf1***, ***Syk***, *Was*, ***Rac2***, *Vav1*
Staphylococcus aureus infection	8	0.001716	*Krt19*, ***Itgam***, *Selplg*, *Krt18*, *C5ar1*, *Ptafr*, *Itgb2*, *Fpr2*
Osteoclast differentiation	8	0.001716	*Lilra6*, *Csf1r*, ***Ncf1***, ***Syk***, ***Ncf4***, *Acp5*, *Lcp2*, *Pirb*
Yersinia infection	8	0.002325	*Was*, ***Rac2***, *Nlrp3*, *Lcp2*, *Nlrc4*, *Mefv*, *Tlr4*, *Vav1*
Chemokine signaling pathway	9	0.004465	*Hck*, *Cxcl12*, ***Ncf1***, *Was*, ***Rac2***, *Plcb1*, *Vav1*, *Pik3cg*, *Pik3r5*
Natural killer cell mediated cytotoxicity	7	0.004616	***Syk***, *Itgb2*, ***Rac2***, *Ptpn6*, *Lcp2*, *Cd48*, *Vav1*
B cell receptor signaling pathway	6	0.004731	*Lilra6*, ***Syk***, ***Rac2***, *Ptpn6*, *Pirb*, *Vav1*

Genes highlighted in bold are considered key regulators due to their recurrence across multiple enriched pathways, suggesting their potential central role in mediating host responses to CCF exposure.
